# Intermediates in the Protein Folding Process: A Computational Model

**DOI:** 10.3390/ijms11084850

**Published:** 2011-07-29

**Authors:** Irena Roterman, Leszek Konieczny, Mateusz Banach, Wiktor Jurkowski

**Affiliations:** 1 Department of Bioinformatics and Telemedicine, Medical College, Jagiellonian University, Lazarza 16, 31-530 Krakow, Poland; E-Mail: mateusz.banach@uj.edu.pl; 2 Chair of Medical Biochemistry, Medical College, Jagiellonian University, Kopernika 7, 31-034 Krakow, Poland; E-Mail: ucjurkow@cyf-kr.edu.pl; 3 Faculty of Physics, Astronomy, Applied Computer Science, Jagiellonian University, Reymonta 4, 30-059 Krakow, Poland; E-Mail: mbkoniec@cyf-kr.edu.pl

**Keywords:** protein structure, hydrophobicity, divergence entropy, intermediates in protein folding

## Abstract

The paper presents a model for simulating the protein folding process *in silico*. The two-step model (which consists of the early stage—ES and the late stage—LS) is verified using two proteins, one of which is treated (according to experimental observations) as the early stage and the second as an example of the LS step. The early stage is based solely on backbone structural preferences, while the LS model takes into account the water environment, treated as an external hydrophobic force field and represented by a 3D Gauss function. The characteristics of 1ZTR (the ES intermediate, as compared with 1ENH, which is the LS intermediate) confirm the link between the gradual disappearance of ES characteristics in LS structural forms and the simultaneous emergence of LS properties in the 1ENH protein. Positive verification of ES and LS characteristics in these two proteins (1ZTR and 1ENH respectively) suggest potential applicability of the presented model to *in silico* protein folding simulations.

## 1. Introduction

Experimental observations of the protein folding process suggest the presence of intermediates [1]. An *in silico* process involving two intermediate steps, accordant with experimental observations, is extensively discussed in [2]. Verification of the model appears possible since A. Fersht has described (in detail) the structural form of the early intermediate of a specific protein belonging to the group of fast-folding proteins [3]. Our two-stage model, consisting of an early stage (ES) and a late stage (LS), is applied to the structure of 1ENH (a type of *Drosophila melanogaster* Engrailed homeodomain [4]), and its L16A mutant 1ZTR [3], treated as the early-stage intermediate (based upon experimental examination) of the 1ENH protein (LS).

According to the presented model, the ES step is assumed to be driven solely by the backbone conformation expressed by two geometric parameters, treated as criteria for structural classification. These two parameters are, respectively, the V-angle (the tilt between two sequential peptide bond planes) and the R-radius of curvature (which appears to be dependent on the V-angle, by way of a second-degree approximation function [5,6]).

The LS model assumes the generation of a tertiary structure centered upon a hydrophobic core. This phenomenon is the consequence of the folding process occurring in a water environment. Generation of a hydrophobic core (*i.e*., migration of hydrophobic residues towards the center of the protein body, with simultaneous exposure of hydrophilic residues on its surface) proceeds in parallel to the standard optimization of internal nonbonding interactions [2]. The analyzed proteins are very good examples of the verification of the model. Unfortunately only a limited number of examples experimentally proven as the early stage of folding process are available. Thus, the verification of the model based on a single protein (in its ES and LS forms) is possible using practically just this two proteins of crystalline structures of the early- and late-stage folding intermediate.

### Materials and Methods

**Two-step model.** The presented model transforms the following folding process:

$$U\;\to {\;I}_{1}\to {\;I}_{2}\to \ldots \ldots \ldots \ldots .\to {\;I}_{n}\to \;N$$

where U—unfolded, I—intermediate the number of which is unknown (possibly dependent on the specificity of the protein molecule), N—native form—into the following:

U → ES → LS → N

where ES and LS denote the early and late stage respectively.

**Figure 1.**
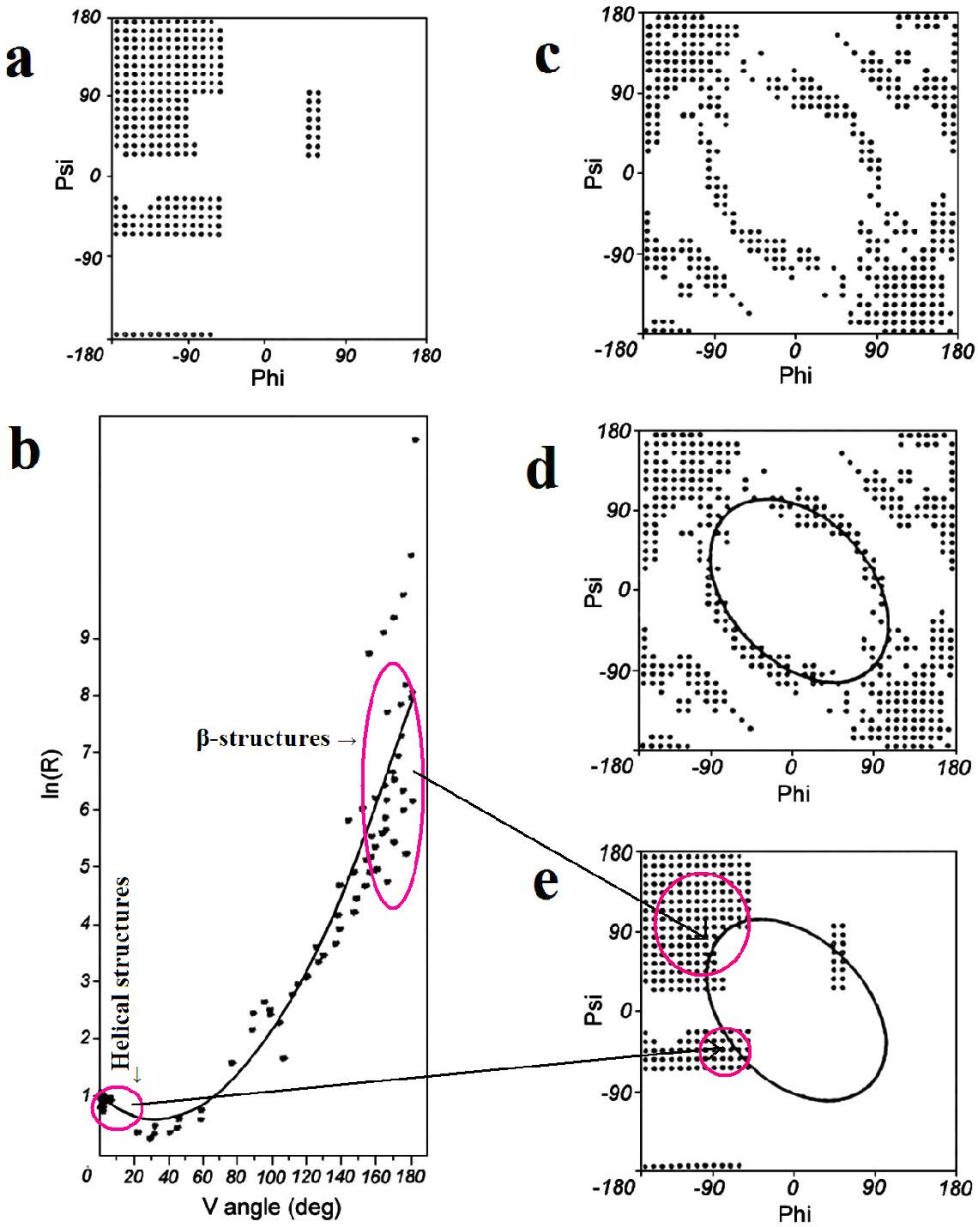
The ES model definition. (**a**) the Ramachandran plot with its low-energy area distinguished; (**b**) the relation between the V-angle (dihedral angle between two sequential peptide bond planes) and R-radius of curvature (using a logarithmic scale to avoid large values for β-structural forms), calculated for structures corresponding to low-energy areas on the Ramachandran plot (shown in a), together with the approximation function (2nd degree polynomial); (**c**) the Ramachandran plot with points representing structures accordant with the approximation function shown in (b); (**d**) the elliptical path assumed to represent the limited conformational subspace for the early-stage intermediate; (**e**) the elliptical path encompassing all secondary structures. The areas distinguished in (b) (small values of V for helical structures and large values for β-like structures) and their placement on the Ramachandran plot (e). The arrows linking b and e show the helical and β-like structures in two representations: V/R parameters (b) and Ramachandran map (e).

**Early stage model.** This model assumes the dominant role of a backbone whose conformation is expressed by two geometric parameters. The first one is the V-angle—the dihedral angle between two sequential peptide bond planes, the value of which is close to 0° for helical forms and close to 180° for extended and β-like structures. The latter, which appears dependent on the former, is the radius of curvature of the polypeptide fragment (calculated for pentapeptide fragments), which is small for helical structures and large for β-structural forms. The relation between these two parameters can be expressed by an approximation function in the form of a second-degree polynomial. According to this model all structures present in proteins may be treated as helixes with a variable radius of curvature—this includes the extended form where the radius of curvature is very large (theoretically infinite). The relation between the V-angle and R-radius of curvature (plotted using a logarithmic scale, to avoid large values for near-planar forms) is presented in Figure 1B.

The values of the V-angle and R-radius of curvature, calculated for pentapeptide fragments and plotted on a coordinate system, enables measurement of the accordance between the assumed model and actual proteins. The average distance between the expected and observed position of a particular point was taken as a criterion of accordance. A value of *D_averaged_* lower than 1 unit indicates good agreement between the model and corresponding observations.

The protein 1ZTR is taken in this paper as the example of ES intermediate according to the experimental observations [3]. If the model is applied for simulation of the folding process, the ES structure is generated on the basis of probability profile along the ellipse path treated as limited conformational sub-space for early steps of folding. This probability profile can be received using the Phi, Psi angles as they appear in PDB (nonredundant database) using the shortest distance criterion. This profile is characterized by seven probability maxima. Some of them represent secondary structural motifs (one maximum-right handed helix, two β-structural and one left-handed helix). Three others represent the unordered structural forms. The recognition of particular maxima—and in consequence recognition of particular structural form (although limited only to the recognition of the structures belonging to ellipse path)—is possible using the contingency table which expresses the relation between sequence of tetrapeptides and their representation in form as defined for ES intermediate. The detailed presentation of this procedure and the ES structural form recognition is given in [5–7].

**Late-stage model.** The tertiary structure of the protein in the LS step of the protein folding process is assumed to involve the generation of a hydrophobic core, together with simultaneous optimization of all other non-bonding interactions (electrostatic, van der Waals and torsion potential) [2]. The presence of an external force field is expressed by a three-dimensional Gauss function. The force field stimulates the hydrophobic core in the “fuzzy oil drop” model directing hydrophobic residues toward the center of the ellipsoid with simultaneous exposure of hydrophilic residues toward the surface (hydrophobic level close to zero) according to the following Gauss function:

$$\tilde{H}t_{j}={\frac{1}{\tilde{H}t}}_{\mathrm{sum}}\text{exp}\left(\frac{-{\left(x_{j}-\bar{x}\right)}^{2}}{2\sigma_{x}^{2}}\right)\text{exp}\left(\frac{-{\left(y_{j}-\bar{y}\right)}^{2}}{2\sigma_{y}^{2}}\right)\text{exp}\left(\frac{-{\left(z_{j}-\bar{z}\right)}^{2}}{2\sigma_{z}^{2}}\right)$$

where *x̄*, *ȳ*, *z̄* are the coordinates of the geometric center of the molecule (usually located at the origin of the coordinate system, where each value can be considered equal to zero). The size of the molecule is expressed by the triple σ_x_, σ_y_, σ_z_, which is calculated for each molecule for each axis (direction) individually, provided that the longest possible distance between interacting atoms along each plane coincides with the appropriate coordinate system axis. σ values are calculated as 1/3 of the longest distance between two effective atoms along each axis. The value of the Gauss function at any point of the protein body is treated as the idealized hydrophobicity density, shaping its hydrophobic core.

Idealized hydrophobicity at any point of the “fuzzy oil drop” can be calculated according to the Gauss function for the molecule whose geometric center lies at the origin of the coordinate system. The empirical hydrophobicity distribution is calculated according to the function presented by Levitt [8]

$$\tilde{H}o_{j}={\frac{1}{\tilde{H}o}}_{\mathrm{sum}}\sum_{i=1}^{N}(H_{i}^{r}+H_{j}^{r})\{\begin{array}{l} \left[1-\frac{1}{2}\left(7{\left(\frac{r_{ij}}{c}\right)}^{2}-9{\left(\frac{r_{ij}}{c}\right)}^{4}+5{\left(\frac{r_{ij}}{c}\right)}^{6}-{\left(\frac{r_{ij}}{c}\right)}^{8}\right)\right]\\ 0\;\text{for}\;r_{ij}\;>c \end{array}\text{for }r_{ij}\;\leq c$$

where *N* expresses the number of amino acids in the protein (number of grid points), ${\tilde{H}}_{i}^{r}$ expresses the hydrophobicity of the *i-th* residue according to the accepted hydrophobicity scale, *r_ij_* expresses the distance between the *i-th* and *j-th* interacting residues and *c* expresses the cutoff distance, which, according to the original paper, is assumed to be 9 Å. The values of *H̃o_j_* are standardized by dividing them by the *H̃o_sum_* coefficient, which is the sum of all hydrophobicities attributed to grid points. The Aboderin scale was applied for calculation [9]. The dependence of the final results on the hydrophobicity scale was presented in details in [10].

A protein whose hydrophobicity distribution is highly consistent with idealized values is treated as structurally accordant with the presented model.

**Kullback-Leibler divergence entropy.** The accordance between the idealized and the observed hydrophobicity distribution is measured according to the Kullback-Leibler relative (divergence) entropy [11], which quantifies the distance between two distributions:

$$D_{KL}(p|p^{0})=\sum_{i=1}^{N}p_{i}{\text{log}}_{2}(p_{i}/p_{i}^{0})$$

where *D_KL_*—distance entropy, *p*—probability of a particular event actually being observed (O), *p*^0^—probability of the same event in the reference distribution (theoretical one denoted as T). The index *i* denotes a particular amino acid while N denotes the number of amino acids in the polypeptide chain. The value of *p* in the equation corresponds to hydrophobicity density attributed to a specific effective atom.

In order to ensure notational uniformity throughout the paper, the above equation can be expressed as:

$$O/T=\sum_{i=1}^{N}O_{i}{\text{log}}_{2}\left(O_{i}/T_{i}\right)$$

The distance between the observed and the theoretical (O/T) values is calculated as the sum (number of residues) of O (observed distribution) *versus* T (theoretical distribution). The symbol T is substituted by *R* taking the random distribution as the reference one.

The distance between both distributions (assuming T and R as a reference) has been calculated for both presented proteins. Entropy can only be interpreted only in form of relative values. Thus the comparison of O/T *versus* O/R may describe the status of particular hydrophobicity distribution. The relation O/T < O/R was taken as evidence for the hydrophobic core accordant with the “fuzzy oil drop” model while the opposite relation O/T > O/R suggests rather random distribution.

## 2. Results and Discussion

In this section, the L16A mutant of the *Drosophila melanogaster* Engrailed homeodomain will be referred to as ES (1ZTR) while its corresponding WT form is assumed to represent the LS (1ENH) structure.

### 2.1. ES Intermediate

**Figure 2.**
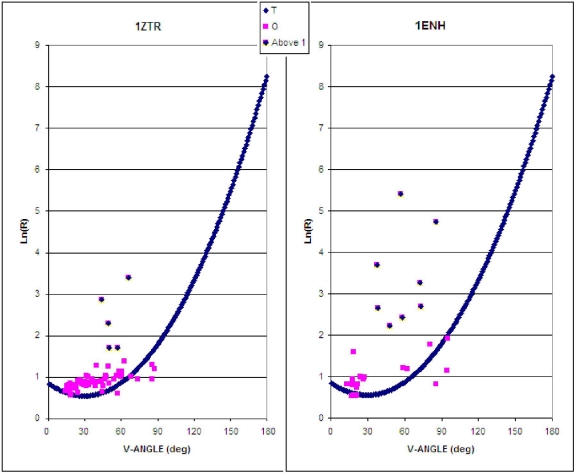
The early stage (ES) model, as applied to 1ZTR (left) and 1ENH (right). Dark blue symbols represent the approximation function, pink squares represent the parameters as they appear in proteins, while dark distributed points residues which differ by more than 1 unit (along the Y-axis).

The characteristics of the ES intermediate and the native structure (treated as the LS intermediate) based on the presented ES model are shown in Figure 2. Assuming an idealized relation between the V-angle and R-radius of curvature, the location of points expressing the geometric parameters of the ES and the native structural form reveals the degree to which both structures are consistent with the assumed model (Figure 2) This accordance can be expressed as higher agreement of assumed model in ES intermediate and its gradual disappearance in LS model (higher values of *D_averaged_* for LS intermediate).

A significantly greater number of residues geometrically accordant with the ES model can be observed in 1ZTR (ES intermediate). The ES protein is treated as a “frozen” early-stage structural form. Poor agreement with theoretical values occurs in the case of 1ENH—it would appear that this protein forfeits its early-stage geometric properties in the process of folding.

**Figure 3.**
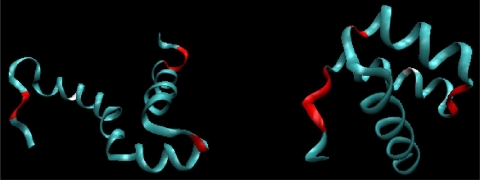
3D structure (ribbon) of 1ZTR (left) and 1ENH (right), with residues represented by dark distributed points in Figure 2. The red fragments represent polypeptides whose positions differ from expectations. The white fragments represent the position of point mutation L16A in ES form (left picture).

The location of fragments accordant with (and divergent from) the assumed model is shown in Figure 3, for both proteins. The values of *D_average_* in Table 1 express the degree of structural changes.

Residues exhibiting significant discrepancies between actual and predicted positions (ln(R), dependent on the V-angle) are located in close proximity to PRO (4) and GLY (39) (PDBSum indices for 1ZTR) in both proteins. The positional irregularity of these residues and their neighbors is probably due to their high structural specificity. PRO is the most rigid residue (φ angle fixed) while GLY is the most flexible residue among the 20 amino acids.

### 2.2. LS Model

The accordance of the tertiary protein structure is measured by comparing the idealized hydrophobicity distribution with empirically observed values. Both proteins were analyzed to enable comparison between early- and late-stage structural forms with respect to the assumed model.

**Figure 4.**
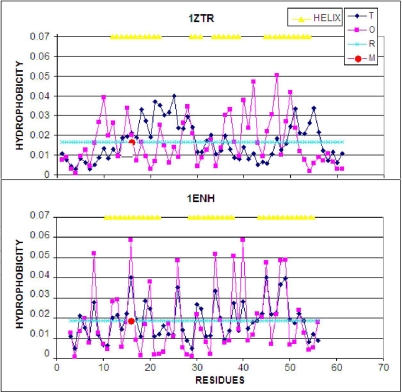
LS model. Top: the hydrophobicity profile plotted along the polypeptide chain in 1ZTR; bottom: the corresponding profile in 1ENH (LS). Dark rhombuses–theoretical distribution; pink squares–observed distribution; light blue line–random distribution. The red circle marks the point of mutation. T, O and R denote the theoretical, observed and random distributions, respectively. The yellow triangles distinguish the helical fragments.

**Figure 5.**
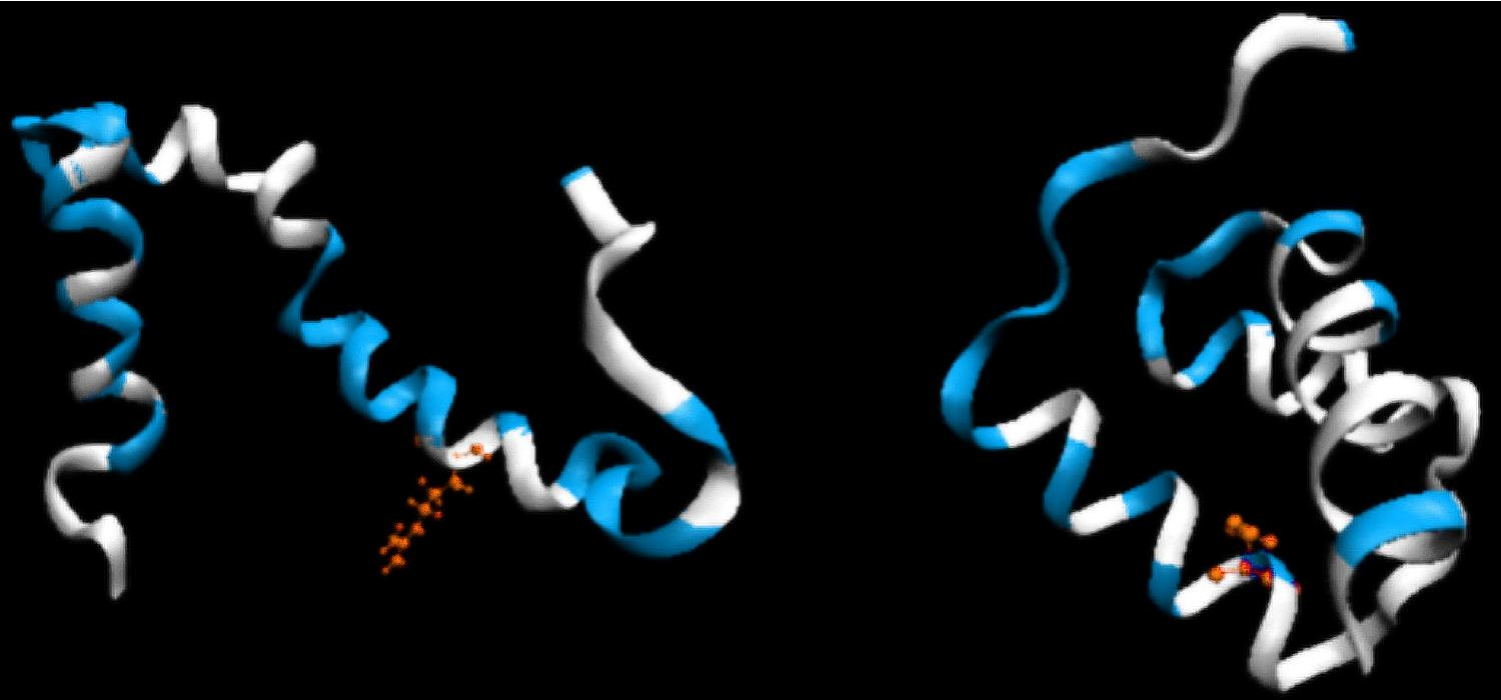
3D representation (ribbon) of 1ZTR (left-ES) and 1ENH (right-LS), with residues whose *D_KL_* values are below 0.01 (good agreement between the predicted and observed hydrophobicity distribution) marked in white. Red residues indicate the point of mutation.

The hydrophobicity distribution (plotted along the polypeptide chain) for both proteins is shown in Figure 4. Visual analysis suggests significantly higher accordance of the hydrophobic core structure in 1ENH (LS form) compared to 1ZTR (ES form). A quantitative comparison is presented in Table 1. The values of O/T and O/R, enabling comparative analysis, are given in Table 1. Significantly better agreement between the observed and idealized hydrophobicity distribution occurs in the LS structural form. The 3D distribution of low- and high-accordance residues is presented in Figure 5.

The presence and role fragment of well defined secondary structure can be seen on profiles presented in Figure 4. The amphiphilic character of helices in 1ENH is visualized as zigzag form of the profile. Almost every one or two residues represent opposite hydrophobic character. The improper (in respect to hydrophobic core structure) orientation of helical fragments in 1ZTR is changed in 1ENH where the accordance between expected and observed distribution is high. The hydrophobicity distributions in the same helical fragments (of amphiphilic character) in both structures do not fit to the expected distribution in the ES structure. It suggests the proper orientation of helices in respect to the hydrophobic core structure in LS intermediate.

## 3. Conclusions

The geometric characteristics of the ES intermediate seem to represent a structural form dominated by backbone preferences which are not evident in the LS structural form. The accordance of the LS model is opposite in character. Poor agreement between the idealized and observed hydrophobicity distributions can be observed in the ES intermediate, while a well-constructed hydrophobic core emerges in the LS intermediate. The loss of idealized backbone geometry on the one hand, with simultaneous generation of a hydrophobic core on the other hand yield hints as to the nature of the folding process.

Both models were positively verified using 1ENH and 1ZTR as examples, in order to estimate the applicability of the presented concept to protein folding simulations. The generation of a tertiary structure based on a hydrophobic core seems to follow theoretical predictions.

The geometric interpretation of the ES structural form carries some consequences related to the definition of a limited conformational subspace of the ES intermediate (Figure 1). Structures which satisfy the proposed relation between the V-angle and R-radius of curvature belong to specific areas on the Ramachandran plot, suggesting an elliptical conformational subspace for the early-stage intermediate. This limited subspace, which appears to be balanced with respect to the amount of information carried by the amino acid sequences and the amount of information necessary to define the protein’s structure, enables generation of starting structures for various optimization methods. Proteins folded with the use of ES intermediates (according to this model) are discussed in [12].

The influence of an external force field representing water and its impact upon the folding process seems to be very well described by a 3D Gauss function. The set of compounds whose structure appears accordant with theoretical predictions includes downhill proteins [13], antifreeze proteins [14] and certain other proteins with varying biological properties. The structure of proteins folded *in silico* in the presence of an external force field was found to be in good agreement with observations [2]. Whenever the shape of the hydrophobic core diverged from the idealized “fuzzy oil drop” model, ligand presence was usually responsible. Protein folding in the presence of an external hydrophobic force field is presented in [14–16], suggesting practical applicability of the model (although the degree of accordance is not yet deemed satisfactory). Structural analysis of trans-membrane proteins in their dynamic forms strongly suggests the reliability of the proposed model [17].

The validity of the assumed model with respect to the influence of an external force field on protein structure suggests a search for other external elements directing the folding process toward the generation of highly specific ligand (substrate) binding sites, as hinted by simulations of ribonuclease [18] and hemoglobin [19], with and without the presence of ligands in the folding environment. Residues characterized by hydrophobicity deficiency (*versus* the idealized distribution) appear to be involved in biological functions such as ligand (substrate) binding [20,21].

Restricting the presented analysis to a pair of proteins was necessitated by the lack of a larger base of experimentally verified early intermediates. An extensive study of the presence of ES characteristics in crystal (native) structures of proteins classified according to the SCOP database can be found in [22]. Proteins classified according to their secondary structure reveal significant representation of ES properties even in LS forms. It seems that significant changes occur in loop fragments (as observed in both proteins discussed in this paper), which calls for the preparation of suitable starting structures for structure prediction algorithms. The conformational subspace defined in this paper helps solve this problem by introducing a method facilitating the search for starting structures.

In conclusion, we can state that:

1. Limiting the conformational subspace for early folding stages seems to be accurate, as proven by the experimentally-verified structure of 1ZTR.
2. The LS step may be simulated through the generation of a hydrophobic core (using a 3D Gauss function), which results in the highest concentration of hydrophobicity at the center of the protein body with simultaneous exposure of hydrophilic residues on the protein surface.
3. The generation of a hydrophobic core (triggered by an external force field) should be taken as an accompanying procedure in the course of internal energy minimization.

The database shows that when proteins fold, a significantly large percentage of nonpolar groups are exposed and a large percentage of charged and polar groups are buried in the interior (groups with opposite charges are usually in contact with each other). This observation appeared to be related to our model. The irregularity of hydrophobic profile appeared to be specific for particular protein. This is why the quantitative measurements of these irregularities was used as the criteria for the recognition of biological function of particular protein. The application and detailed analysis is presented in previous papers [10,20].

## Figures and Tables

**Table 1.** O/T and O/R parameters for 1ZTR and 1ENH, treated as ES and LS (native) structural forms respectively. O/T denotes the distance between observed (O) and theoretical (T) distribution which is used as a reference, while O/R denotes the distance between observed (O) and random (R) distribution. The relation O/T < O/R is treated as an indicator of agreement between the observed structure of the hydrophobic core and the assumed “fuzzy oil drop” model.

	**ES MODEL**	**LS MODEL**	

**PROTEIN**	***D_average_***	**O/T**	**O/R**
**1ZTR (ES structure)**	0.342	0.4978	0.3638
**1ENH (LS structure)**	0.623	0.1286	0.2137
